# Effects of Transcutaneous Auricular Vagus Nerve Stimulation on the P300: Do Stimulation Duration and Stimulation Type Matter?

**DOI:** 10.3390/brainsci14070690

**Published:** 2024-07-10

**Authors:** Manon Giraudier, Carlos Ventura-Bort, Mathias Weymar

**Affiliations:** Department of Biological Psychology and Affective Science, Faculty of Human Sciences, University of Potsdam, Campus Golm, Karl-Liebknecht-Str. 24/25, 14476 Potsdam, Germany; ventura@uni-potsdam.de (C.V.-B.); mathias.weymar@uni-potsdam.de (M.W.)

**Keywords:** tVNS, taVNS, EEG, P300, noradrenaline, salivary alpha-amylase, stimulation parameters

## Abstract

Non-invasive transcutaneous auricular vagus nerve stimulation (taVNS) has attracted increasing interest as a neurostimulation tool with potential applications in modulating cognitive processes such as attention and memory, possibly through the modulation of the locus–coeruleus noradrenaline system. Studies examining the P300 brain-related component as a correlate of noradrenergic activity, however, have yielded inconsistent findings, possibly due to differences in stimulation parameters, thus necessitating further investigation. In this event-related potential study involving 61 participants, therefore, we examined how changes in taVNS parameters, specifically stimulation type (interval vs. continuous stimulation) and duration, influence P300 amplitudes during a visual novelty oddball task. Although no effects of stimulation were found over the whole cluster and time window of the P300, cluster-based permutation tests revealed a distinct impact of taVNS on the P300 response for a small electrode cluster, characterized by larger amplitudes observed for easy targets (i.e., stimuli that are easily discernible from standards) following taVNS compared to sham stimulation. Notably, our findings suggested that the type of stimulation significantly modulated taVNS effects on the P300, with continuous stimulation showing larger P300 differences (taVNS vs. sham) for hard targets and standards compared to interval stimulation. We observed no interaction effects of stimulation duration on the target-related P300. While our findings align with previous research, further investigation is warranted to fully elucidate the influence of taVNS on the P300 component and its potential utility as a reliable marker for neuromodulation in this field.

## 1. Introduction

Transcutaneous auricular vagus nerve stimulation (taVNS) has gained increasing interest as a non-invasive neuromodulation technique to affect the activity of the vagus nerve and its associated brain circuits, offering potential benefits for the treatment of a wide range of clinical disorders (for review, see [1]), including chronic pain [2], depression [3], and pharmacoresistant epilepsy [4,5,6]. In non-clinical populations, taVNS has also shown promise in modulating various cognitive and affective processes, such as attention, cognitive control [7,8,9], learning and memory [10,11,12,13,14], and emotion recognition [15].

One hypothesized mechanism through which taVNS may exert its effects is the modulation of the locus coeruleus–noradrenaline (LC-NA) system (for review, see [16]). The afferent fibers of the vagus nerve transmit bodily information, including adrenergic release, from the adrenal gland to the brain [17]. This information is received by the nucleus tractus solitarii (NTS) [18,19], which subsequently sends excitatory signals to the nucleus paragigantocellularis (PGi) [20]. The PGi, in turn, projects to the LC-NA system [21,22], which further sends projections to various brain regions through an extended neuronal network, including frontal and medio-temporal regions [23]. Animal studies have provided initial evidence for the modulatory role of the vagus nerve on LC-NA system activity, reporting increased LC-firing rates following invasive vagal nerve stimulation [24,25,26,27,28] and reduced firing after vagotomy [29]. In animals, invasive vagus nerve stimulation (iVNS) has further demonstrated an improvement in processes mediated via the LC-NA system, such as extinction learning [30,31], memory consolidation [32], and inhibitory avoidance learning [33]. Similar findings have been reported in humans, with some studies showing improvement in verbal recognition memory ([34,35], but see [36]). Further evidence comes from animal and human studies relating vagal activity to physiological markers of LC-NA system activity, particularly in terms of phasic activity [28,37,38,39]. Phasic activity refers to the rapid, transient bursts of neural activity that occur in response to salient stimuli. These bursts of activity are associated with changes in physiological markers of LC-NA system activity, including pupil dilation and the P300 event-related potential (ERP) component [37] (for review, see [22,40]). Studies in rodents have shown an increase in LC activity and pupil size during iVNS [28,39]. Additionally, the P300, also known as P3, manifests as a positive deflection in electroencephalographic (EEG) signals, primarily observed in centro-parietal areas and typically occurring within the 300–600 ms window after the stimulus onset. It is elicited as a result of the attentional response to infrequent target stimuli, which are presented within a series of more frequent non-target standard stimuli, a setup commonly referred to as the oddball paradigm. The P300 component is modulated through various psychological states, including the tonic arousal level [41], the perceived relevance of the stimulus [42], the subjective likelihood of its occurrence [43,44], and the cognitive resources dedicated to stimulus processing [45,46,47]. At a neurophysiological level, the generation of this component has been suggested to reflect the phasic activity of the neuromodulatory LC-NA system [22,37] in response to unexpected target stimuli requiring an immediate behavioral response [37,40,48]. In previous iVNS studies, increased P300 amplitudes have been observed in epileptic patients, as well as increased P300 amplitudes in depressive patients [49], indicating potential modulation of the LC-NA system [38].

In light of the existing evidence supporting the modulatory effects of invasive vagal stimulation on LC-NA system activity, particularly evident in animal and clinical human studies, recent research has sought to examine whether non-invasive taVNS elicits comparable effects on LC-NA activity in healthy humans. Studies using brain imaging have initially confirmed enhanced functional LC activation during taVNS compared to sham stimulation in healthy participants [50,51,52,53,54,55], suggesting that the effects of taVNS may follow the same anatomical pathway as iVNS. Furthermore, several studies have investigated the modulatory effect of taVNS on various cognitive and affective processes that are associated with noradrenergic signaling. Positive effects of taVNS have been reported in the context of fear extinction [10,56,57], memory [11,12,13,14], cognitive control [8,15,58,59], and attention [7,9]. However, some studies have also reported no significant effects of taVNS on these processes (for fear extinction, see [56,60]; for memory, see [36,61]; for cognitive control, see [62]; for attention, see [63]).

Similarly, studies investigating the impact of taVNS on physiological markers of phasic LC-NA system activity have also yielded inconsistent findings (for review, see [64]). The modulatory effects of taVNS on pupil dilation [65,66,67,68,69,70] have not been consistently replicated [59,63,71,72,73], and studies on the effects of taVNS on the P300 amplitude have also produced mixed results. While some studies have reported an increase of the parietal P300 amplitude during taVNS compared to sham stimulation in auditory [7] and visual [74,75,76] oddball tasks (for at least a subset of stimuli), others have reported no significant effects [8,71,77,78]. These mixed results could potentially be attributed to the variability in stimulation procedures, including differences in stimulation parameters (e.g., stimulation type, referring to the distinction between continuously delivered stimulation and stimulation delivered in intervals, stimulation frequency, and pulse width) and stimulation duration (for review, see [16]). Further elucidating the stimulation parameters and circumstances under which the effects of taVNS-mediated vagal activation are more pronounced concerning correlates of LC-NA activity is crucial for optimizing the effectiveness of taVNS interventions in clinical and research settings, potentially leading to more consistent outcomes and a better understanding of its action mechanisms [66,79,80].

In the current study, therefore, we aimed to investigate how parametric modifications of stimulation type and stimulation duration modulate the taVNS effects on the P300 ERP component using a novelty oddball task [75] (while the stimulation frequency and pulse width were fixed at 25 Hz and 200–300 μs, respectively, these parameters can vary between studies; however, their optimal settings for P300 ERP modulation had not been tested so far). In Ventura-Bort et al. [75], taVNS, compared to sham stimulation, was found to increase the P300 amplitude only for easy targets (i.e., stimuli that are easily discernible from standards) relative to standard stimuli, thus providing preliminary evidence for a modulatory influence of taVNS on the P300 (for easy targets) using a novelty oddball task. Notably, larger differences between taVNS and sham stimulation in P300 amplitudes for easy targets were associated with larger increases in salivary alpha-amylase (sAA) levels after taVNS but not after sham stimulation. In order to address some limitations of the pilot study conducted by Ventura-Bort et al. [75], we adopted a randomized, single-blinded, taVNS–sham, within-subject, cross-over design with a larger sample of participants who completed the same visual novelty oddball task with an increased number of trials. Following the assumption that the LC-NA system modulates the parietal P300, we expected to observe an interaction between stimulus type (targets vs. standards) and stimulation (taVNS vs. sham), exclusively in the easy condition, replicating previous findings [75]. In addition to the interacting effects of taVNS, in the current study, we further investigated whether such effects were modulated via the stimulation type (continuous vs. interval stimulation) and stimulation duration during the experimental block (i.e., the temporal extent of a taVNS application) [81,82].

## 2. Materials and Methods

### 2.1. Participants

A total of 70 healthy students (55 female and 15 male, $M_{age}=23.93,SD_{age}=4.15$) participated in the study for course credits or financial compensation. However, 9 participants were excluded from the analyses due to technical difficulties or missing data (i.e., study dropout), leaving a final sample of 61 participants (48 female and 13 male, $M_{age}=23.65,$ $SD_{age}=4.08$). Out of this sample, 31 participants were randomly assigned to receive continuous stimulation (24 female and 7 male, $M_{age}=24.29,SD_{age}=4.26$), while the other 30 participants received interval stimulation (24 female and 6 male, $M_{age}=23,SD_{age}=3.78$). All participants were German speakers (at least C1 level) and had normal or corrected-to-normal vision. Exclusion criteria were neurological or psychiatric disorders, brain surgery, undergoing medication or drug use, pregnancy, a history of migraine and/or epilepsy, cardiac diseases, metal pieces in the body (e.g., a pacemaker), and active implants or physical alterations in the ear (e.g., a cochlear implant). An a priori power analysis was conducted to determine the required sample size, f=0.17,α=0.05,power=0.99. The analysis estimated a sample of 66 participants for this study. The study was pre-registered on the Open Science Framework (https://osf.io/tbwq9/, accessed on 10 March 2023).

### 2.2. Experimental Procedure

A 2-day, randomized, single-blinded, taVNS–sham, within-subject, cross-over design was applied to investigate how the stimulation duration and stimulation type may modulate the interacting effects of taVNS on the P300. In each session (seven days apart), participants were randomly assigned to receive continuous or interval taVNS or sham stimulation in alternating sequences throughout the study sessions, ensuring a balanced distribution across all conditions.

**Figure 1:**
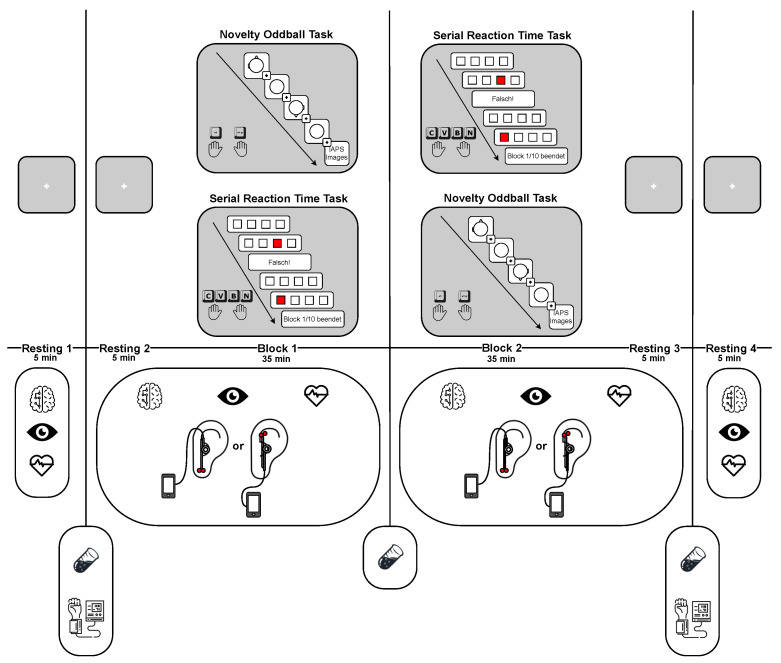
Schematic overview of the experimental procedure in each session, depicting task presentation (novelty oddball task and serial reaction time task, upper part) and measured variables, as well as the stimulation condition (lower part) with corresponding time points. Symbols represent the following measurements and conditions: electroencephalography (EEG) is indicated with a brain symbol, electrocardiography (ECG) with a heart symbol, oculography with an eye symbol, and saliva collection for salivary alpha-amylase (sAA) analysis with a saliva sample symbol. Blood pressure measurement is represented with a blood pressure device symbol. The stimulation condition (taVNS vs. sham) is indicated with a corresponding symbol. Both stimulation conditions were applied to all participants on two separate days, one week apart.

Participants began each session with a 5 min resting phase without any stimulation in a sound-attenuated, dimly lit experimental room, during which baseline measurements of resting state (electroencephalography, electrocardiography, and oculography) were taken. Following this initial resting phase, participants received 5 min of stimulation without any tasks. Subsequently, participants were instructed to perform two tasks consecutively in block 1: a novelty oddball task [75] and a serial reaction time task [83]. Stimulation was administered concurrently during these tasks. Following the completion of the first block, participants immediately proceeded to the second experimental block, which included the same tasks. Stimulation continued throughout the second block. Each block lasted 35 min. After completing the two experimental blocks, participants entered a 5 min resting phase with stimulation still ongoing. Following this resting phase, the stimulation was turned off, and participants had a fourth 5 min resting phase without any stimulation (see Figure 1).

Systolic and diastolic blood pressure, along with sAA levels, were measured immediately before and after the stimulation. Additionally, sAA levels were measured between blocks (i.e., after 40 min of stimulation). At the end of the experiment, participants completed a questionnaire on stimulation side effects, for which they had to indicate on a 7-point scale (1 being not at all and 7 being very much) how much they had experienced headache, nausea, dizziness, neck pain, muscle contractions in the neck, stinging sensations under the electrodes, skin irritation in the ear, fluctuations in mental concentration or feelings, and other unpleasant feelings or adverse effects. It should be noted that the results of the resting phases and of the serial reaction time task will be reported elsewhere.

### 2.3. Transcutaneous Auricular Vagus Nerve Stimulation (taVNS) Procedure

Electrical stimulation of the vagus nerve was conducted using two titan electrodes attached to a mount and wired to a stimulation unit (NEMOS; tVNS Technologies GmbH, Erlangen, Germany). In the vagus condition, the stimulator electrodes were placed on the left cymba conchae, an area exclusively innervated via the auricular branch of the vagus nerve [84,85]. In the sham condition, the electrodes were positioned on the left earlobe, an area known to be free of vagal innervation [84,85]. The stimulation parameters were used as predefined for the NEMOS device with a frequency of 25 Hz and a pulse width of 200–300 μs. Stimulation was delivered for a total of 80 min. To explore the effects of stimulation duration, we defined two distinct temporal patterns of taVNS application during the experimental blocks. Specifically, short-duration stimulation refers to the first block, while long-duration stimulation corresponds to the second block. Additionally, to assess the impact of different stimulation types, participants received either continuous stimulation throughout the experimental procedure or interval stimulation, with alternating on and off phases every 30 s. Following the protocol by Ellrich et al. [85] (see also, e.g., [13,75]) stimulation intensity was adjusted individually for each participant above the detection threshold and below the pain threshold.

### 2.4. Rotated-Heads Novelty Oddball Paradigm

Participants completed a modified version of the rotated-heads oddball task [86]. The task included three types of stimuli: standards, targets, and novel stimuli. Standard stimuli were presented in 70% of the trials (n = 336) and consisted of a plain oval. Target stimuli, comprising a schematic head created by combining the oval with a nose and an ear, were presented in 15% of the trials (n = 72). Participants were instructed to indicate whether the ear was positioned on the left or right side of the nose by pressing the corresponding response button. The target trials were split into two categories: in half of the trials (n = 36), the nose pointed upwards, making it easy to identify the ear’s position (easy condition). In the other half of the trials (n = 36), the nose was pointing down, requiring participants to mentally rotate the head (hard condition). This differentiation allowed us to assess the impact of taVNS on the P300 ERP component for both types of targets, providing insights into the differential effects of parametric modifications on task performance and neural responses. Novel stimuli were further presented in 15% of the trials (n = 72) and were composed of two sets, each consisting of 72 emotional (overall 48 neutral, 48 unpleasant, and 48 pleasant) images selected from the International Affective Picture System [87]. The two sets were matched for their emotional and physical attributes and were presented in alternating order across sessions.

Stimuli were presented for 100 ms, with a variable inter-trial interval between 1500 ms and 3000 ms. The order of stimuli was pseudo-randomized with the restriction that no more than two non-frequent (target/novel) stimuli were presented successively. Prior to the actual task, participants performed 20 practice trials (50% standards and 50% targets). Stimulus presentation and data recording were managed using the Presentation software version 20.0 (Neurobehavioral Systems Inc., Berkely, CA, USA).

### 2.5. Electrophysiological Recording

EEG signals were recorded from a 129-channel HydroCel Geodesic Sensor Net (Magstim EGI, Eugene, OR, USA) using NetStation software version 5.4.2 on an Apple Macintosh Computer (Cupertino, CA, USA). EEG signals were continuously sampled at a rate of 250 Hz and referenced online to the vertex (Cz). The scalp impedance for each sensor was kept below 50 kΩ and below 5 kΩ for the reference electrode. The EEG data were analyzed offline using BrainVision Analyzer version 2.3.0 (Brain Products GmbH, Gilching, Germany). To reduce power-line noise at 50 Hz and stimulation noise at 25 Hz, and to potentially improve independent component analysis (ICA) performance [88,89], all channels were band-pass filtered from 0.1 Hz to 20 Hz. Visual inspection was used to identify and interpolate any bad channels, and the data were converted to the average reference [90]. ICA was then performed to remove (ocular) artifacts. Stimulus-synchronized epochs were extracted from 200 ms before to 800 ms after stimulus presentation. Separate ERP averages were computed for each condition and block of every participant in each of the experimental conditions, i.e., standard, easy target, and hard target. Given previous results observing taVNS effects primarily for the parietal P300 in response to target stimuli [75], our analysis focused solely on the contrast between target and standard conditions, excluding comparisons involving novel stimuli.

Due to the insufficient number of good trials left after EEG-data preprocessing and the persistence of high levels of noise in the recorded data, EEG data from 14 participants were excluded. Analysis proceeded with the remaining 47 participants. The noise in the EEG data was believed to be caused either by the stimulation or by the eye tracker (since the majority of the noise was found in the area around both ears), which resulted in artifacts that were difficult to remove during the preprocessing stage without potentially removing important effects (e.g., by using band rejection). Hence, we decided to exclude these participants to ensure that the results were not influenced by the remaining noise and artifacts, thus enhancing the reliability and accuracy of the analysis.

### 2.6. Statistical Analyses

Statistical analyses were conducted with R version 4.3.0 (R Core Team, 2023). The level of statistical significance was set to 0.05.

#### 2.6.1. Stimulation Intensity

To examine differences in stimulation intensities, *t*-tests were performed, comparing taVNS and sham stimulation both overall and within the continuous stimulation and interval stimulation conditions.

#### 2.6.2. Stimulation Side Effects

To examine any potential adverse effects resulting from the stimulation, separate *t*-tests for each reported subjective symptom were performed, comparing taVNS and sham stimulation. Furthermore, a MANOVA was computed to explore whether participants who received continuous stimulation exhibited side effects differently compared to those who received interval stimulation.

#### 2.6.3. Behavioral Performance

Behavioral performance was assessed for accuracy and response time, using repeated-measures ANOVAs, including the within-subject factors target (easy vs. hard), stimulation (taVNS vs. sham), stimulation duration (short-duration vs. long-duration stimulation), and between-subject factor stimulation type (continuous vs. interval stimulation). Trials with incorrect responses or with response times below 150 ms (i.e., anticipatory responses) or above 1500 ms (i.e., misses) were discarded (8.07% of trials).

#### 2.6.4. Electrophysiological Data

**Figure 2:**
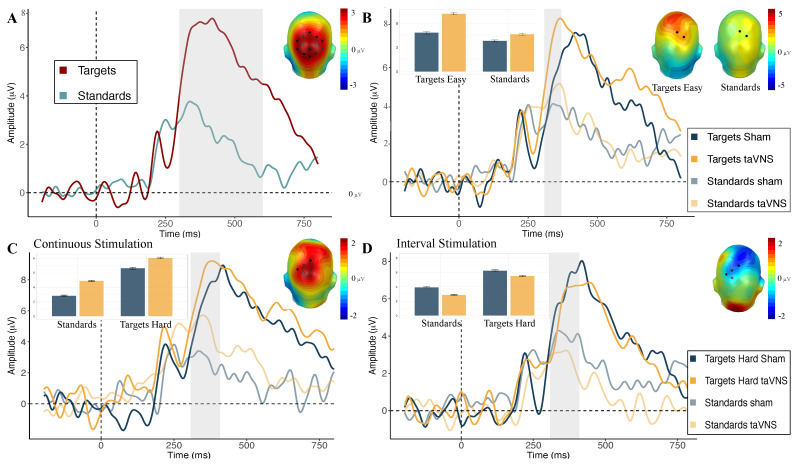
(**A**) General P300 grand average event-related potential (ERP) in the 300–600 ms time window (highlighted in gray) over a parietal electrode cluster (E53, E54, E55, E60, E61, E62, E67, E72, E77, E78, E79, E85, and E86) for targets and standards. Scalp topography of the ERP difference between targets and standards is depicted in the inset. (**B**) Grand average ERPs showing the differential effects of stimulation (taVNS vs. sham) over a significant parietal electrode cluster (E55 and E79) for easy targets and standards. Notably, the significant effect of stimulation was found only for easy targets, not for standards. Scalp topographies of the ERP differences between taVNS and sham are depicted in the insets, with the significant time window (308–368 ms) highlighted in gray. Barchart depicting mean amplitudes for stimulus type (targets easy vs. standards)under taVNS (orange) and sham stimulation (blue) conditions. (**C**) Grand average ERPs showing the differential effects of stimulation (taVNS vs. sham) over a significant parietal electrode cluster (E53, E54, E55, and E61) for hard targets and standards for continuous stimulation and (**D**) for interval stimulation. Notably, the significant effect between stimulation and stimulation type was consistent for hard targets and for standards. Scalp topographies of the ERP differences between taVNS and sham are depicted in the insets, with the significant time window (308–408 ms) highlighted in gray. Bar charts depicting amplitudes by stimulus type (targets hard vs. standards) for continuous stimulation and interval stimulation under the taVNS (orange) and sham stimulation (blue) conditions. See Figure A2 for an additional bar chart not differentiating by stimulus type and a corresponding table with means and standard deviations.

To analyze the effects of stimulation on the P300, we used a two-step procedure. First, based on previous research [41,91,92] and on visual inspection, we identified the cluster and time window in which the P300 was maximal (i.e., where the difference activity target vs. standards was more positive). Importantly, the cluster, as well as the time window, were blindly selected without any knowledge about the potential effects of the stimulation (ERPs were averaged across stimulation conditions). The P300 was identified in the 300–600 ms time window over a parietal electrode cluster (E53, E54, E55, E60, E61, E62, E67, E72, E77, E78, E79, E85, and E86) (see Figure 2A). In a second step, a cluster-based permutation test [93] was performed using the matlab-based EMEGS software version 2.8 [94] on the selected time window and parietal cluster. The use of the cluster-based permutation test allowed us to effectively address the trade-off between false positive and false negative outcomes, thereby optimizing the detection of true effects and enhancing reproducibility [93,95]. This methodological approach comprises a two-step procedure to identify significant effects among different experimental conditions. Initially, F-tests were performed for each time point and sensor. Clusters were identified when statistical significance (p≤0.05) persisted for a minimum of five consecutive time points (equivalent to 20 ms) and involved at least five neighboring sensors. The F-values within these identified clusters were aggregated into cluster masses. Subsequently, Monte Carlo simulations encompassing 1000 permutations of experimental conditions and participants were executed. Random permutation cluster masses were generated and juxtaposed against the original cluster masses (p=0.05). Only cluster masses surpassing the specified alpha level within a predefined time epoch and electrode site were considered statistically significant.

To evaluate the effects of taVNS on brain dynamics (i.e., target processing), the cluster-based permutation tests were run on the effects of repeated-measured ANOVAs with the within-subject factors stimulus type (easy vs. standards and hard vs. standards), stimulation (taVNS vs. sham), stimulation duration (short-duration vs. long-duration stimulation), and the between-subject factor stimulation type (continuous vs. interval stimulation). As the effects of taVNS are assumed to differently affect both target categories (easy vs. hard) (see [75]), ANOVAs were carried out separately for both target categories (easy target processing and hard target processing).

To further validate the necessity of the chosen liberal approach, a traditional ANOVA analysis was conducted on the P300 ERP component across the predefined parietal electrode cluster (E53, E54, E55, E60, E61, E62, E67, E72, E77, E78, E79, E85, and E86) during the time interval of 300–600 ms (see Figure 2A). This conventional analysis, albeit widely used, failed to reveal significant effects associated with the experimental conditions (see Table A1 and Table A2 for detailed results). Such null findings underscore the limitations of traditional methods when exploring complex neural dynamics, particularly in scenarios where effects may be subtle or distributed across multiple electrodes and time points.

#### 2.6.5. Hormonal Data

The effects of stimulation on sAA levels were investigated using a repeated-measure ANOVA including the within-subject factors time (pre vs. post), stimulation (taVNS vs. sham) and the between-subject factor stimulation type (continuous vs. interval stimulation). To further investigate the association between changes in sAA levels (post vs. pre) and P3 amplitudes, correlation analyses were separately conducted using electrophysiological data recorded within the 300–600 ms time window over the specified parietal electrode cluster for easy target and hard target processing.

## 3. Results

### 3.1. Stimulation Intensity

The individually adjusted stimulation intensity varied from 0.5 mA to 2.5 mA for both continuous and interval stimulation ($M_{continuous}=1.39$, $SD_{continuous}=0.49$, $M_{interval}=1.24$, and $SD_{interval}=0.56$) in the vagus condition. For the sham condition, the continuous stimulation varied from 0.8 mA to 3.3 mA ($M_{continuous}=1.98$ and $SD_{continuous}=0.65$), and the interval stimulation varied from 0.6 mA to 4.0 mA ($M_{interval}=1.80$ and $SD_{interval}=0.70$). Stimulation intensities did not differ significantly between the groups for taVNS, t(59)=−1.07, p=0.29, or for sham stimulation, t(59)=−1.02, p=0.31. Within the groups, stimulation intensities differed significantly, indicating overall higher intensities for sham stimulation compared to taVNS in the continuous stimulation condition, t(60)=−3.97, p=0.0002, and in the interval stimulation condition, t(58)=−3.36, p=0.001.

### 3.2. Stimulation Side Effects

Participants’ subjective ratings revealed minimal side effects associated with the stimulation (continuous stimulation: N=31, M=2.28, SD=1.25; interval stimulation: N=30, M=2.03, SD=1.31; see Table 1). Statistical analyses indicated no significant differences between taVNS and sham stimulation for any of the assessed side effects (*p*s >0.06). However, participants receiving continuous stimulation reported significantly higher levels of dizziness, F(1,118)=4.81, p<0.05, neck pain, F(1,118)=8.23, p<0.01, neck contraction, F(1,118)=6.29, p<0.05, and decreased concentration, F(1,118)=3.92, p<0.05, irrespective of whether they received taVNS or sham stimulation. Other side effects did not differ between continuous and interval stimulation (*p*s >0.2). It should be noted that the highest rating for any side effect was 3.97 on a scale ranging from 1 (indicating not at all) to 7 (indicating very much), suggesting overall low levels of side effects.

### 3.3. Behavioral Performance

**Figure A1:**
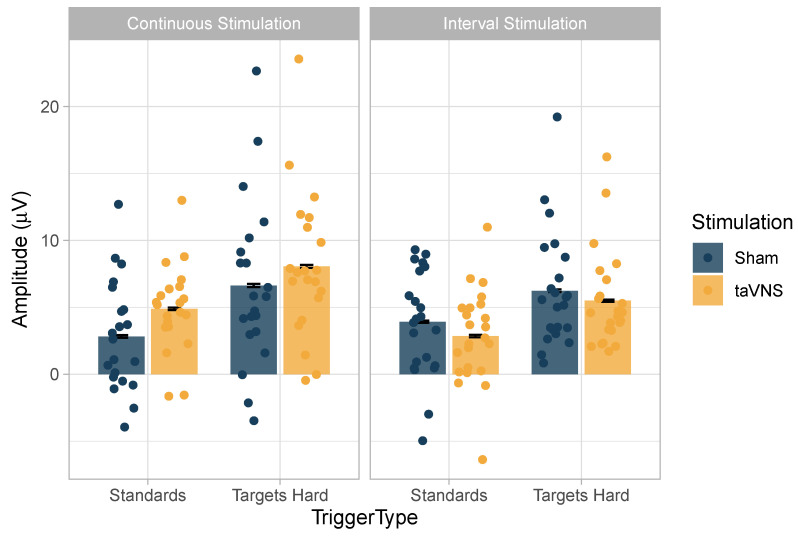
Bar chart depicting response times for targets (easy vs. hard) for short-duration stimulation and long-duration stimulation under the taVNS (orange) and sham stimulation (blue) conditions. The bars represent the mean response times, while the individual data points indicate the distribution of response times.

The behavioral performance revealed no significant main effects (Fs<1) and no significant interactions in terms of accuracy. For response times, a significant main effect for target revealed faster response times for easy compared to hard targets, F(1,59)=316.91, p<0.001, $\eta_{p}^{2}=0.84.$ No other main effects were observed. Furthermore, a significant interaction between target and stimulation duration, F(1,59)=4.66, p<0.05, $\eta_{p}^{2}=0.07$, showed faster response times for hard targets in long-duration stimulation compared to short-duration stimulation, t(121)=2.28, p=0.02, whereas response times for easy targets were identical for short-duration and long-duration stimulation, t(121)=0.44, p=0.66. The interaction between the stimulation, stimulation duration, and target was also significant, F(1,59)=6.83, $p=0.01,\eta_{p}^{2}=0.10$, showing faster response times for hard targets during long-duration stimulation compared to short-duration stimulation in the taVNS condition, t(60)=3.56, p<0.001, but not in the sham condition, t(60)=−0.10, p=0.92 (see Figure A1), suggesting that only participants receiving taVNS significantly improved in terms of response times over time (short-duration vs. long-duration stimulation) when responding to hard targets. See Table A3 and Table A4 for detailed behavioral performance results.

### 3.4. Electrophysiological Data

#### 3.4.1. Easy Target Processing

**A cluster-based permutation test revealed a stimulus-type effect in a classical P300 time window.** In the classical P300 time window of 300–600 ms, one parietal cluster (sensors: E53, E54, E55, E60, E61, E62, E67, E72, E77, E78, E79, E85, and E86; mass: 23,262) was found that exceeded the threshold for cluster correction (critical cluster mass: 162.5). The cluster extended from 304 to 604 ms and revealed a main effect of stimulus type, F(1,42)=37.83; p<0.001, with larger ERP amplitudes toward easy targets compared to standards ($M_{TargetsEasy}=5.98,\;SD_{TargetsEasy}=7.41;\;M_{Standards}=2.24,\;SD_{Standards}=6.65$).

**Cluster-based permutation test revealed stimulation effects and interacting effects with stimulation duration but no interacting effects for stimulation type.** The cluster-based permutation test further revealed a main effect of stimulation, F(1,42)=6.52; p=0.014, as indicated with a significant cluster (sensors: E55, E78, E79, E85, and E86; mass: 304.9) extending from 308 to 376 ms (critical cluster mass: 158.5), which reflected enhanced amplitudes for taVNS compared to sham stimulation ($M_{taVNS}=5.64,\;SD_{taVNS}=6.95;$ $M_{sham}=4.25,$ $SD_{sham}=8.22$). Interestingly, the cluster-based permutation test found a significant cluster (sensors: E53, E60, E61, E62, and E67; mass: 176.8) between 452 and 488 ms that exceeded the threshold for cluster correction (critical cluster mass: 163) and revealed a significant interaction between the stimulation, stimulation duration, and stimulus type, F(1,42)=4.16; p=0.048. This interaction was further investigated by decomposing it into two separate ANOVAs, one for the easy targets and another for the standard stimuli. Notably, cluster-based permutation tests for each revealed that the effect of stimulation only appeared for the easy targets, F(1,42)=7.42; p=0.009, as indicated with a significant cluster (sensors: E55 and E79; mass: 169.4) extending from 308 to 368 ms (critical cluster mass: 165), but not for the standard (critical cluster mass: 134). The significant main effect indicated greater activity in the taVNS condition compared to the sham stimulation condition for easy targets ($M_{taVNS}=7.22,\;SD_{taVNS}=7.62;\;M_{sham}=4.85,\;SD_{sham}=8.81$) (see Figure 2B). In contrast, a significant cluster emerged (sensors: E53, E54, E55, E61, E62, E77, E78, E79, E85, and E86; mass: 2407.1) for the standard stimuli for the 304–576 ms (critical cluster mass: 179.5) time window that revealed a significant main effect of stimulation duration, F(1,45)=7.43; p=0.009. This significant main effect was reflected by larger ERP amplitudes for short-duration stimulation compared to long-duration stimulation for standard stimuli ($M_{short-duration}=3.43,\;SD_{short-duration}=6.57;\;M_{long-duration}=1.70,$ $SD_{long-duration}=6.56$).

#### 3.4.2. Hard Target Processing

**A cluster-based permutation test revealed a stimulus-type effect in the classical P300 time window.** In the classical P300 time window of 300–600 ms, a cluster was identified (sensors: E53, E54, E55, E60, E61, E62, E67, E72, E77, E78, E79, E85, and E86; mass: 23,272.8) that surpassed the threshold for cluster correction (critical cluster mass: 143), between 312 and 604 ms. The cluster demonstrated a significant main effect of stimulus type, F(1,42)=39.24; p<0.001; specifically, larger ERP amplitudes were observed for hard targets in comparison to standard stimuli within this parietal cluster ($M_{TargetsHard}=5.95,$ $SD_{TargetsHard}=7.10;\;M_{Standards}=2.22,\;SD_{Standards}=6.66$).

**A cluster-based permutation test revealed an interacting effect of stimulation with the stimulation type but no interacting effects of stimulation duration.** The cluster-based permutation test comparing the differential effects of stimulation on hard target processing over parietal regions further revealed a cluster exceeding the critical cluster mass of 179. The significant cluster (sensors: E53, E54, E55, and E61; mass: 479.8) was found between 308 and 408 ms, and it indicated a significant interaction between stimulation and the stimulation type, F(1,42)=9.11; p=0.004, showing higher overall taVNS-driven P300 amplitudes for hard targets and standards under continuous stimulation (taVNS vs. sham), *t*(20) = 2.69, *p* = 0.01, but not under interval stimulation (taVNS vs. sham), t(22)=−1.52, p=0.14. The results also revealed significant differences for taVNS under continuous stimulation compared to interval stimulation (continuous taVNS vs. interval taVNS), t(38.84)=−2.38, p=0.02, but not for the sham condition (continuous sham vs. interval sham), t(39)=0.33, *p* = 0.74, further enhancing the robustness of the observed effect between stimulation and the stimulation type (see Figure 2C,D).

### 3.5. Hormonal Data

The hormonal data revealed no significant main effects (Fs≤1) and no significant interactions (stimulation × time, F(1,59)=2.75, p=0.10, stimulation × stimulation type, F<1, time × stimulation type, F(1,59)=1.64, p=0.20, stimulation × time × stimulation type, F(1,59)=2.63, p=0.11). Correlation analyses further revealed that the increase in sAA levels (post vs. pre) did not correlate with the P300 amplitudes for taVNS, r(42)=0.03, p=0.84 or correlate with the P300 amplitudes for sham stimulation, r(42)=−0.06, p=0.71, in easy target processing and hard target processing, respectively (for taVNS, r(42)=−0.03, p=0.86, for sham stimulation, r(42)=0.14, p=0.38).

## 4. Discussion

In the present study, we explored how changes in taVNS parameters, specifically stimulation type and duration, affected the P300 as a potential physiological marker of LC-NA activity (for review, see [64]). Cluster-based permutation tests performed on the P300 component (i.e., a predefined time window and cluster within P300 activity) revealed effects of stimulation that were partly in line with previous research [75]. Specifically, our results revealed a stimulus-specific effect of taVNS on the P300 response, showing larger P300 amplitudes for easy targets after taVNS compared to sham stimulation in a small set of electrodes. Notably, our findings also suggested that the type of stimulation significantly modulated taVNS effects on the P300, with continuous stimulation showing larger P300 differences (taVNS vs. sham) for hard targets and standards compared to interval stimulation. Given the differential effects of taVNS on easy targets compared to hard targets, the potential mechanisms underlying these distinct responses are discussed separately.

### 4.1. Easy Target Processing

Studies have provided rather inconsistent evidence for an effect of taVNS on P300 amplitudes [64]. Whereas some studies found an increase of P300 amplitudes during taVNS compared to sham stimulation [7,48,74,76], others failed to observe such an enhancement effect [8,71,77,78]. Notably, Ventura-Bort et al. [75] observed a taVNS-related enhancement in P300 amplitude specifically during a visual novelty oddball task. This effect was discerned solely through a post hoc exploratory analysis using easy targets, rather than an overall difference (easy and hard targets) between taVNS and sham stimulation. Consistent with these findings [75], our results similarly suggest that taVNS modulated P300 brain activity primarily for easy targets, indicating a stimulus-specific effect (i.e., larger P300 amplitudes for easy targets after taVNS compared to sham stimulation). Specifically, when breaking down the triple interaction between stimulation (taVNS vs. sham), stimulation duration (short-duration vs. long-duration stimulation), and stimulus type (easy targets vs. standards) into two distinct analyses for standard and easy target stimuli, we found that the effects of stimulation were significant solely for easy targets, with no impact observed for standards (in a small set of electrodes). Notably, the processing of hard targets did not reveal a main effect of stimulation, as will be discussed later on. One potential explanation, also proposed in Ventura-Bort et al. [75], is that the cognitive processes involved in mental rotation when processing hard targets (which is not necessary for the processing of easy targets) may obscure the impact of taVNS on P300 amplitudes. Indeed, mental rotation tasks require engagement of spatial working memory [96,97] and are associated with changes in brain activity (e.g., over central parietal regions from 350 to 800 ms after the stimulus onset; [98,99]) potentially overlapping with the neural processes underlying the P300 response. This overlapping activity might obscure the beneficial effects of taVNS on P300 amplitudes. On a behavioral level, our findings align with the notion of increased cognitive demands during mental rotation tasks (i.e., higher reaction times for hard targets compared to easy targets) [100]. Further investigation, however, is needed to elucidate the specific influence of mental rotation tasks on the neural processes underlying the P300 response in the context of taVNS.

In addition to the interacting effects of taVNS on P300 amplitudes during easy target processing, in the current study, we further investigated whether such effects were modulated via the stimulation type and stimulation duration. Our results did not show any effects of the stimulation type on the P300 during easy target processing, suggesting that the stimulation type might not be that relevant, at least for this type of target processing. Unexpectedly, our analysis revealed that stimulation duration influenced P300 amplitudes for standard stimuli but not for easy targets, independent of the stimulation condition (taVNS vs. sham). The decrease in P300 amplitudes over time for standards suggests that there are differences in cognitive processing or attentional allocation. Participants might have been more alert, attentive, or engaged at the beginning of the experiment and, over time, habituated to the task demands. Standard stimuli, being more frequently presented throughout the task, may have elicited greater habituation effects over time compared to easy target stimuli, which were less common and potentially more salient, maintaining participants’ attention and cognitive engagement consistently. Indeed, Geisler and Polich [101] have previously reported such decreases in P300 amplitudes for standard stimuli across trial blocks while noting no such decrease for target stimuli. Extending this result, we also found that the duration of taVNS had no effect on the processing of standard stimuli (or target stimuli) over time.

### 4.2. Hard Target Processing

When evaluating the effects of taVNS on hard target processing (hard targets vs. standards), interestingly, we found that the stimulation type did modulate the effects of taVNS on the P300 response. Specifically, we found that continuous, compared to interval, taVNS led to higher overall P300 amplitudes for hard targets and standards. This suggests that continuous stimulation may be more effective than interval stimulation when processing hard targets and standards. There is some evidence pointing towards an advantage for continuous stimulation, as opposed to interval stimulation, the latter of which might lead to a rapid decline in NA activity, potentially attenuating the modulatory effects of taVNS for markers of noradrenergic activity. This is consistent with animal studies showing that such a decline in NA occurred after invasive vagal stimulation was turned off [39,102]. A recent analysis reported an advantage for continuous stimulation over interval stimulation when pooling raw data from a large sample of taVNS studies that collected sAA levels as a potential marker of central NA release [103]. Animal models have further substantiated these findings, showing decreased NA levels after invasive vagal stimulation was turned off [39,102]. The observed advantage of continuous stimulation over interval stimulation in processing both hard targets and standards may be attributed to their shared characteristic of requiring action suppression, distinguishing them from easy targets. While standard stimuli do not require mental rotation like hard targets, participants still need to suppress any impulse to respond when presented with a standard stimulus. Since these stimuli do not require a response based on the position of the ear relative to the nose, participants must inhibit any prepotent response tendencies. Similarly, participants encountering hard targets face the challenge of mentally rotating the schematic head to determine the ear’s position. This cognitive process requires suppressing the impulse to respond. In contrast, easy targets provide a clear and straightforward task, and there is no need for action suppression, as opposed to hard targets and standards. Action suppression, implicated in inhibitory control processes, has been associated with the P300 component, suggesting a relationship between the P300 amplitude and the motor output in the context of inhibitory control [104,105]. This indicates that P300 may reflect the varying engagement of inhibitory control. Several lines of evidence further suggest the modulation of inhibitory control processes via the noradrenergic system (and potentially the GABAergic system; see [106,107]). Increased NA concentrations in the prefrontal cortex have been associated with improved response inhibition performance in both rodents and humans [108,109,110], including individuals with attention deficit hyperactivity disorder [108]. The NA system may also affect backward inhibition processes and the impact of working memory load on response inhibition processes, which implies that NA modulation could influence response inhibition not only directly but also indirectly through its effects on working memory, as working memory processes are also influenced via the NA system [111,112]. Considering that taVNS is thought to exert its effects via NA modulation, it is conceivable that taVNS, particularly continuous taVNS (as opposed to interval taVNS, which might lead to a rapid decline in NA activity), might influence the suppression of action processes for hard targets and standards. On a behavioral level, our results align with a similar trend indicating that participants receiving taVNS show improvement over time when processing hard targets (i.e., reduced response times), suggesting a parallel effect of taVNS on the suppression of action processes. Continuous taVNS may, thus, enhance inhibitory control processes, potentially through its effects on noradrenergic activity, leading to improved cognitive performance. Additionally, previous research indicates that taVNS enhances response selection processes, particularly under high selection demands [83]. This finding aligns with our observed improvements in reaction times and P300 amplitudes during hard target processing, further supporting the role of taVNS in enhancing action control. Interestingly, for behavioral data, stimulation duration appears to be a critical component, whereas for the electrophysiological data, specifically the P300 component, the stimulation type might play a more prominent role (i.e., no effects of stimulation duration on the target-related P300 were observed during hard target processing), highlighting the differential impact of stimulation parameters across neurophysiological and behavioral levels of analysis. It needs to be noted, however, that a recent meta-analysis was unable to demonstrate an effect of stimulation duration on behavioral performance across multiple studies [80]. This discrepancy might be attributed to the heterogeneity in stimulation parameters collected in the meta-analysis. Alternatively, it is possible that variability in stimulation duration is a less critical factor in driving cognitive changes than the stimulation itself. Therefore, the influence of taVNS parameters such as stimulation duration warrants further investigation.

### 4.3. Hormonal Response

The current study also collected sAA data as an additional physiological marker of LC-NA system activity. While some studies investigating the effects of taVNS on sAA levels have reported increased sAA levels post-stimulation compared to sham stimulation [71,75,103], suggesting sAA as a potential marker of central NA enhancement mediated via taVNS, others found no effects [12,72,73,78,82,113]. In our recent mega-analysis, pooling raw data from numerous taVNS studies that collected sAA levels as a potential marker of central NA release, we found an effect of taVNS compared to sham stimulation and attributed the inconsistency and lack of replicability reported across taVNS studies regarding sAA levels to relatively small sample sizes within individual studies and methodological heterogeneity between studies [103]. Notably, in the Ventura-Bort et al. [75] study, primary analyses did not reveal any effects of stimulation, but direct testing indicated that taVNS, and not sham stimulation, increased sAA levels. Furthermore, larger differences between taVNS and sham stimulation in P300 amplitudes for easy targets were associated with greater increases in sAA levels after taVNS, but not after sham stimulation. However, our study failed to replicate these findings, as we found no correlations between sAA levels (post vs. pre) and P300 amplitudes for taVNS compared to sham, during both easy and hard target processing. Indeed, it is conceivable that differences in study characteristics, including experimental designs, stimulation procedures, methodological differences in data collection, preprocessing, and statistical analysis, contribute to the inconsistent findings across studies, including our own. It is worth noting that, while our study employed the same task as Ventura-Bort et al. [75], there were notable differences in our experimental setup (e.g., different types of stimulation were administered, our participants engaged in the novelty oddball task twice, a longer task and experimental duration were used, and another task was included).Furthermore, methodological differences were evident in our analyses; for instance, we used a logarithmic transformation of the typically skewed sAA data [114]. All of these differences might provide an explanation for not replicating the effects of taVNS on sAA levels. Additionally, it has been previously suggested that the observed effect of vagal stimulation on sAA levels might be relatively small [103], which could further elucidate why this effect was not observed in the current single-study design. This needs, however, to be further investigated in future studies.

### 4.4. Limitations

Our study addressed important limitations of Ventura-Bort et al. [75] by adopting the same visual novelty oddball task while expanding the trial count and participant pool. While the current results are partly consistent with previous findings [75], indicating a stimulus-specific effect of taVNS on P300 modulation (i.e., for a predefined time window and small electrode cluster within P300 activity) and, additionally, differential modulatory effects of stimulation type, our study also highlights the complexity of interpreting the P300 component as an LC-NA marker in taVNS research. The involvement of various cognitive processes in the visual novelty oddball task, such as mental rotation and inhibitory control processes, suggests that the P300 response may reflect a multitude of underlying mechanisms, likely beyond noradrenergic activity alone (for review, see [115]). Furthermore, our findings should be considered in light of the potential limitations due to the complexity of our experimental design, making it difficult to attribute observed effects solely to the influence of taVNS on noradrenergic activity. The interplay between task demands, stimulation parameters, and individual differences adds further complexity to the interpretation of our results. Additionally, a significant amount of ERP data was lost due to poor data quality, which emphasizes that our findings have to be interpreted with caution and need further verification. Consequently, we opted to employ a cluster-based permutation test specifically within a predefined P300 time window and electrode cluster. Although analysis across a broader electrode set revealed a notable triple interaction effect among stimulation, stimulation duration, and the stimulus type, discernible enhancement specific to taVNS in response to easy targets was observed only in a small subset of electrodes (see Figure 2B). Thus, further investigation is warranted to elucidate the precise role of the P300 component in taVNS research and its potential as a reliable marker for neural modulation. Future studies might also focus on alternative stimulation conditions, such as phasic, event-related stimulation (e.g., for review, see [116]).

## 5. Conclusions

Despite these limitations, our study contributes valuable insights into the effects of taVNS on neural and physiological markers of noradrenergic activity. Our findings align with a stimulus-specific effect of taVNS on P300 modulation, with significant effects observed primarily for easy targets. The stimulation type (continuous vs. interval stimulation) did not affect P300 amplitudes during easy target processing, while an advantage of continuous stimulation over interval stimulation in processing hard targets and standards was observed, suggesting a relationship between continuous taVNS and its facilitation of putative action suppression processes. No effects of stimulation duration were observed on the target-related P300. Further research is needed to fully understand the influence of taVNS parameters on the P300 component and its potential as a reliable marker for neuromodulation.

## Figures and Tables

**Table 1:** Mean subjective rating (standard deviation) for the continuous and interval stimulation side effects in the taVNS and sham conditions for continuous and interval stimulation. Ratings were scored on a seven-point scale with 1 being not at all and 7 being very much.

	Continuous Stimulation			Interval Stimulation		
	taVNS	Sham	* p * -Value	taVNS	Sham	* p * -Value
Headache	2.00 (1.24)	2.19 (1.38)	0.47	2.13 (1.41)	2.23 (1.57)	0.72
Nausea	1.16 (0.45)	1.26 (0.57)	0.45	1.23 (0.93)	1.13 (0.57)	0.63
Dizziness	1.64 (0.98)	1.48 (0.72)	0.41	1.33 (0.71)	1.20 (0.48)	0.38
Neck pain	3.03 (1.58)	3.35 (1.85)	0.29	2.13 (1.33)	2.60 (1.54)	0.06
Neck contraction	3.42 (1.57)	3.48 (1.48)	0.82	2.60 (1.71)	2.80 (1.85)	0.41
Stinging sensation	2.03 (1.47)	2.16 (1.61)	0.66	2.33 (1.58)	2.10 (1.67)	0.46
Ear irritation	1.84 (1.39)	1.55 (1.15)	0.29	1.47 (0.94)	1.40 (0.93)	0.60
Concentration	3.97 (1.62)	3.68 (1.64)	0.28	3.20 (1.88)	3.20 (1.79)	1
Fluctuations of feelings	1.84 (1.32)	1.87 (1.12)	0.86	1.70 (1.32)	1.90 (1.40)	0.33
Unpleasant feelings	1.87 (0.88)	1.74 (0.96)	0.49	2.03 (1.50)	1.83 (1.18)	0.36

**Figure A2:**
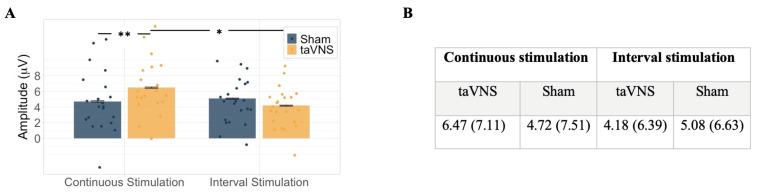
(**A**) Bar chart depicting amplitudes for stimulation type (continuous vs. interval stimulation) under the taVNS (orange) and sham stimulation (blue) conditions, independently of stimulus type. * p<0.05, ** p<0.01. (**B**) Table with means and standard deviations in parentheses.

## Data Availability

All data have been made publicly available on the Open Science Framework and can be accessed at https://osf.io/tbwq9/, accessed on 10 March 2023.

## Appendix A

**Table A1:** Results of a classical ANOVA approach depicting easy target processing within the P300 time window (300–600 ms) across 13 electrodes. Electrodes included in the analysis: E53, E54, E55, E60, E61, E62, E67, E72, E77, E78, E79, E85, and E86. “Stimulation” is abbreviated as “stim”, and ”DoF” stands for “degrees of freedom”.

	DoF	F-Value	*p*-Value	$\eta_{p}^{2}$
**Main effects** Stimulation (Stim.) Stim. Duration Stimulus Type Stim. Type	42 42 42 42	0.304 3.072 37.379 1.653	0.584 0.087 0.000 0.206	7.18 × 10^−3^ 0.07 0.47 0.04
**Two-way interactions** Stim. × Stim. Duration Stim. × Stimulus Type Stim. × Stim. Type Stim. Duration × Stimulus Type Stim. Duration × Stim. Type Stimulus Type × Stim. Type	42 42 42 42 42 42	0.018 0.105 0.652 0.893 1.417 0.631	0.893 0.747 0.424 0.350 0.241 0.431	4.33 × 10^−4^ 2.50 × 10^−3^ 0.02 0.02 0.03 0.01
**Three-way interactions** Stim. × Stim. Duration × Stimulus Type Stim. × Stim. Duration × Stim. Type Stim. × Stimulus Type × Stim. Type Stim. Duration × Stimulus Type × Stim. Type	42 42 42 42	1.504 0.085 1.199 5.794	0.227 0.772 0.280 0.021	0.03 2.03 × 10^−3^ 0.03 0.12
**Four-way interactions** Stim. × Stim. Duration × Stimulus Type × Stim. Type	42	0.017	0.897	4.02 × 10^−4^

**Table A2:** Results of a classical ANOVA approach depicting hard target processing within the P300 time window (300–600 ms) across 13 electrodes. Electrodes included in the analysis: E53, E54, E55, E60, E61, E62, E67, E72, E77, E78, E79, E85, and E86. “Stimulation” is abbreviated as “stim”, and ”DoF” stands for “degrees of freedom”.

	DoF	F-Value	*p*-Value	$\eta_{p}^{2}$
**Main effects** Stimulation (Stim.) Stim. Duration Stimulus Type Stim. Type	42 42 42 42	0.535 0.401 37.795 1.680	0.468 0.530 0.000 0.202	0.01 9.45 × 10^−3^ 0.47 0.04
**Two-way interactions** Stim. × Stim. Duration Stim. × Stimulus Type Stim. × Stimulation Type Stim. Duration × Stimulus Type Stim. Duration × Stim. Type Stimulus Type × Stim. Type	42 42 42 42 42 42	2.140 0.029 1.864 3.852 0.999 0.451	0.151 0.866 0.179 0.056 0.323 0.505	0.05 6.85 × 10^−4^ 0.04 0.08 0.02 0.01
**Three-way interactions** Stim. × Stim. Duration × Stimulus Type Stim. × Stim. Duration × Stim. Type Stim. × Stimulus Type × Stim. Type Stim. Duration × Stimulus Type × Stim. Type	42 42 42 42	0.022 0.145 0.378 0.008	0.882 0.705 0.542 0.928	5.35 × 10^−4^ 3.44 × 10^−3^ 8.92 × 10^−3^ 1.96 × 10^−4^
**Four-way interactions** Stim. × Stim. Duration × Stimulus Type × Stim. Type	42	0.000	0.983	1.10 × 10^−5^

**Table A3:** Mean accuracy (standard deviation) for the continuous and interval stimulation in the taVNS condition and the sham condition, separately for easy and hard targets for short-duration and long-duration stimulation.

	Continuous Stimulation		Interval Stimulation	
	taVNS	Sham	taVNS	Sham
**Short-duration** Easy Hard	97.52 (6.62) 97.16 (6.11)	98.18 (5.31) 98.12 (4.44)	98.20 (3.46) 98.24 (4.92)	98.24 (5.18) 99.02 (2.94)
**Long-duration** Easy Hard	98.25 (3.26) 97.96 (3.59)	97.66 (5.71) 97.56 (6.09)	98.52 (2.28) 99.11 (1.57)	98.04 (3.94) 97.86 (4.35)

**Table A4:** Mean response time (standard deviation) for the continuous and interval stimulation in the taVNS condition and the sham condition, separately for easy and hard targets for short-duration and long-duration stimulation.

	Continuous Stimulation		Interval Stimulation	
	taVNS	Sham	taVNS	Sham
**Short-duration** Easy Hard	642.20 (159.18) 819.75 (202.31)	641.28 (157.29) 813.21 (188.84)	668.48 (158.52) 830.38 (195.05)	653.73 (146.18) 782.46 (181.30)
**Long-duration** Easy Hard	641.60 (155.89) 796.72 (195.17)	650.43 (159.65) 815.79 (202.21)	662.25 (148.42) 796.95 (185.82)	644.54 (146.42) 783.95 (185.67)
